# Association of metabolic syndrome with inflammatory mediators in women with previous gestational diabetes mellitus

**DOI:** 10.1186/2251-6581-12-8

**Published:** 2013-01-22

**Authors:** Banoo Edalat, Farshad Sharifi, Zohre Badamchizadeh, Arash Hossein-Nezhad, Bagher Larijani, Mojde Mirarefin, Hossein Fakhrzadeh

**Affiliations:** 1 Researcher, Elderly Health Research Center, Endocrinology & Metabolism Research Institute, Tehran University of Medical Sciences, Tehran, Iran; 2 Masters of Nutrition, Elderly Health Research Center, Endocrinology & Metabolism Research Institute, Tehran University of Medical Sciences, Tehran, Iran; 3 Associate Professor of Cardiology, Endocrinology and Metabolism Research Center, Tehran University of Medical Sciences, Tehran, Iran; 4 PHD of Geriatric Epidemiology, Tehran, Iran; 5 Bachelor of Nursing, Elderly Health Research Center, Endocrinology & Metabolism Research Institute, Tehran University of Medical Sciences, Tehran, Iran; 6 Assistant Professor of Genetics, Endocrinology & Metabolism Research Institute, Tehran University of Medical Sciences, Tehran, Iran; 7 Professor of Internal Medicine, Endocrinology & Metabolism Research Institute, Tehran University of Medical Sciences, Tehran, Iran

**Keywords:** Inflammation mediators, Diabetes gestational, Metabolic syndrome X

## Abstract

**Background:**

An increased risk of metabolic syndrome (MS) has been observed among women with previous gestational diabetes mellitus (pGDM). Increased inflammatory markers such as C-reactive protein (CRP) and interleukin 6 (IL-6) usually accompany. We performed this survey to examine the relationship between pGDM and MS, CRP and IL-6.

**Methods:**

77 women with pGDM and 67 randomly sampled women free from GDM participated in this study, 2–3 years after index pregnancy. Laboratory and anthropometric measurements were performed. MS was defined according to ATP III criteria. Statistical analyses were conducted using SPSS 18.

**Results:**

CRP were different between groups with and without pGDM [2.69 (2.86 mg/dl and 1.56 (1.39) mg/dl, respectively; *p* < 0.01]. The presence of each MS component by itself was associated with significantly higher CRP Levels, except for fasting blood glucose. In linear regression models, CRP and IL-6 were significantly associated with BMI (β =0. 25, 0.23; *p* < 0.01), waist circumference (β=0. 27, 0.05; *p* < 0.01) and HOMA-IR (β=0. 39, 0.39; *p* < 0.01). After adjustment for age and BMI the occurrence of pGDM in the group with both high CRP and MS was significantly associated with CRP level (OR= 5.11; CI=1.59-16.43; *p* < 0.01).

**Conclusion:**

Since CRP and Il-6 were higher in women with both pGDM and MS it appears that the presence of pGDM with MS components have a synergistic effect on the elevation of serum levels of inflammatory markers which can be partly as a result of visceral obesity. Further long-term studies are necessary to confirm the relationship between CRP, IL-6 and MS in women with pGDM.

## Introduction

Gestational diabetes mellitus is a condition in which blood glucose level increases during pregnancy [1]. Its prevalence in Iranian pregnant women is 4.7% [2]. Although women with gestational diabetes mellitus (GDM) usually revert to normal status after delivery, it has adverse effects on maternal and fetal health [3]. Women with a history of GDM are at significantly increased risk of developing type 2 diabetes mellitus [4,5] which is itself an important risk factor of cardiovascular diseases and the latter is a leading cause of death among these patients [6]. But it seems that GDM-affected women are more prone to metabolic syndrome (MS) too [7,8].

Further understanding of the underlying mechanisms of type 2 diabetes mellitus and its cardiovascular complications may lead to a better prevention and treatment.

Inflammation may be the missing link between pGDM, type 2 diabetes mellitus (DM) and MS, as it is associated with insulin resistance [9]. It has been indicated that increased inflammation is an independent risk factor for the development of type 2 DM [10,11]. It also has adverse effects on the vascular system and plays an important role in increasing cardiovascular risk. Many of these studies have revealed that inflammation is a critical factor in the initiation of atherosclerosis and acute plaque rupture which result in acute heart attack and stroke [12,13].

Recent studies have focused on C-reactive protein (CRP) more than other inflammatory markers. CRP is a protein that is synthesized by liver which is then secreted into circulation. It is a critical component of the immune system and is one of the acute phase proteins that is increased during systemic inflammation. On average, CRP levels tend to rise with an increase in blood pressure, smoking and increment in BMI. On the contrary, thin and athletic subjects have lower levels of CRP. Studies indicate that the higher the levels of circulating CRP, the higher will be the risk of type 2 DM, myocardial infarction, stroke and peripheral vascular disease, too [7]. So, it may be also associated especially with cardiovascular features of MS [8].

In addition some studies have analyzed the role of IL-6 in the atherosclerosis process as this marker has a concordance with CRP in the inflammatory response. It is to say that IL-6 induces CRP production in the liver by activating Janus kinases. Signal transducers and activators of transcription subsequently switch on the CRP gene expression, leading to its production. Interleukin 6 is a single chain protein that is produced by immune cells. It is a cytokine that provokes a broad range of cellular, physiological and immune responses. This cytokine is relevant to diabetes mellitus and acute coronary syndrome, too [14-16].

Due to relatively high prevalence of pGDM in Iranian women and its potential complications, we conducted this survey to find out the association of pGDM and MS with inflammatory markers such as CRP and IL-6 in a sample of women with pGDM compared to non-pGDM ones.

## Methods

### Study population

**Figure 1:**
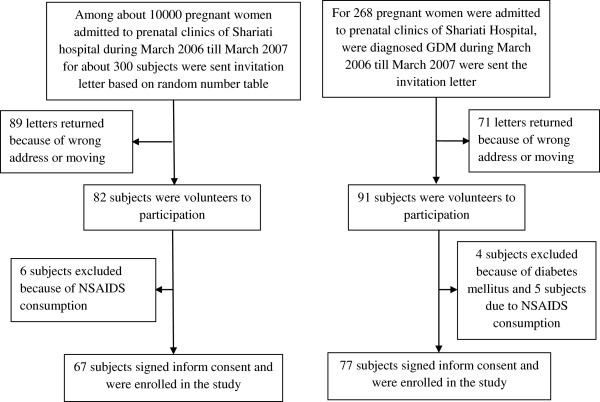
Flow of the participants in case and control groups.

This is a retrospective cohort study which was conducted since March until May 2009. Prospective participants were chosen by recalling pregnant women who had been admitted to prenatal clinics of Shariati Hospital from March 2006 until March 2007. During this period, 268 pGDM women were admitted to these clinics. Invitation letters were sent to all of them. 71 letters were returned due to the wrong address or house moving. 95 subjects volunteered to participate but finally only 77 subjects were recruited for the laboratory tests (Figure 1). Control subjects were chosen from pregnant women attending the prenatal clinics during the same period. Invitation letters were sent to 300 subjects based on a random number table. Only 92 subjects replied and 67 people were recruited to this study (Figure 1).

Inclusion criteria included: having the history of a live delivery during last 24–36 months, being 20–45 years old and having done the 75-gram glucose tolerance test between 24^th^ –26^th^ weeks of gestation.

Women with diabetes antedating pregnancy, the ones who were taking aspirin which could influence CRP levels, having the history of preeclampsia or eclampsia in each of their pregnancies, and also those who had an inflammatory or chronic disease such as rheumatic or chronic infectious disease were excluded.

All of the enrolled women had performed the glucose tolerate test during the index pregnancy, which was done as the measurement of plasma glucose after consumption of 75 g glucose between the 24^th^ and 26^th^ weeks of gestation. The classification as having GDM was according to the World Health Organization (WHO) criteria.

### Clinical measurements

Weight was measured in light clothing to the nearest 0.1 Kg (using an electronic scale) and height was measured without shoes to the nearest 1 cm on a standardized, wall-mounted height board. Waist circumference was measured to the nearest 1 centimeter with a non-elastic flexible tape measure while the subjects were in the standing position at the end of gentle exploration, laterally on the midway between the lowest portion of the rib cage and iliac crest, and anteriorly midway between the xiphoid process of the sternum and the umbilicus [12] Body mass index (BMI) was calculated as the ratio of the weight (kg) to the square of height (m^2^).

Systolic (SBP) and diastolic blood pressures (DBP) were measured two times by a physician using the Automatic device (Omron 7) which had been calibrated by mercury sphygmomanometer according to *The Seventh Report of the Joint National Committee on Prevention, Detection, Evaluation, and Treatment of High Blood Pressure* (JNC7).

### Definition of conditions

The metabolic syndrome was defined according to *The National Cholesterol Education Program’s Adult Treatment Panel III report* (ATP III) criteria [17]. Insulin sensitivity was measured by HOMA-IR using the Mathew’s simplified formula [18]:

(1) $$\text{HOMAIR}\;=\left[\text{fasting insulin mUI/ml}\right]\times \left[\text{fasting glucose mmol/}\;l\right]/22.5$$

The cut-point for diagnosis of high CRP level was defined as CRP≥3 mg/dl [19].

### Biochemical measurements

Blood samples were collected into venoject tubes after an overnight fast (12 hours). The drawn samples were then transferred into EDTA tubes, and immediately placed in an ice box. The plasma was separated by centrifuging the samples at 2000 rpm, which was immediately sent to the biochemical laboratory and stored at - 60°C until being assayed. Glucose was measured using the enzymatic colorimetric method. Total cholesterol, high density lipoprotein, low density lipoprotein and triglycerides were measured by the enzymatic method. These measurements were performed by the auto - analyzer (Hitachi Manneheim 902, Germany). CRP was measured using an ELISA with a sensitivity of .05 mg/L and the intra-assay coefficients of variation of 5%. C-reactive protein values were measured in duplicate, and the average value was reported for both assays. Plasma adiponectin concentrations were measured by a validated sandwich ELISA using an adiponectin-specific antibody. The intra-assay and inter-assay coefficients of variation were 3.8% and 7.9%, respectively. Insulin was measured by radioimmunoassay method (Monobind, USA). Plasma interleukin-6 levels were measured by enzyme linked immunosorbent assay (Cayman, USA). The detectable limit for interleukin-6 was 0.10 pg/ml, and the inter-assay coefficient of variation was 7%.

### Statistical analysis

Statistical analyses were performed using SPSS 18.0; PASW statistics. All variables except for CRP had a normal distribution. So Manne-Whitney U test and medians were used. Data are given as a % or mean ± standard deviation (SD). All statistical comparisons were considered significant if the p values were less than 0.05 ( *p <* 0*.*05). In case of normality in the distribution of variables, the two-tailed Student’s *t*-test was employed to compare two sets of quantitative data. The Chi-square test was used to compare the observed frequency between groups as far as qualitative variables were concerned. Multiple logistic regressions were carried out to evaluate Odds Ratio (OR).

### Ethical considerations

This study was conducted according to the principles outlined in the Declaration of Helsinki. All participants signed a written consent which was approved by the ethical committee of Endocrinology and Metabolism Research Institute (EMRI) affiliated to Tehran University of Medical Sciences. Interpretation of Laboratory and paraclinical data was provided for all participants and if necessary they were referred to a specialist for more management.

## Results

**Figure 2:**
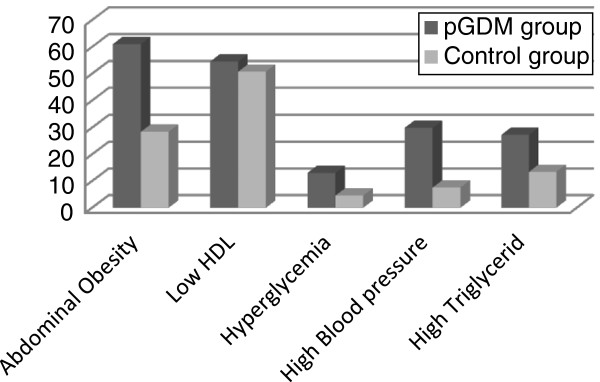
The prevalence (%) of the components of metabolic syndrome in pGDM and control groups.

Mean age of the participants was 32.33 ± 5.46 (31.66 years ± 5.51 and 32.87 years ± 5.40 in control and pGDM groups respectively; p=0.19). The laboratory data and clinical characteristics of the 144 subjects, based on the history of GDM, are shown in Table 1. In a logistic regression model the metabolic syndrome was entered as a dependent variable of pGDM. The odds ratio of metabolic syndrome was 3.68 (1.25-10.70) after adjustment for age and BMI. The most frequent metabolic trait was abdominal obesity (61% in pGDM women and 28.4 in the control group) and the least frequent one was high blood glucose (13% and 4.5%, respectively). Between pGDM women and controls there were not any significant differences in most of the variables studied, except for some components of MS (i.e. waist circumference, TG, systolic and diastolic blood pressure), which were predictable (Figure 2). In comparison with controls, those with pGDM had a more prevalent history of type 2 diabetes mellitus in their family, which was acceptable considering the role of family history in the occurrence of type 2 diabetes mellitus [20]. There was an association between fasting serum insulin and also HOMA-IR with CRP in all the participants (β=0.28, p=0.00 and β=0.35, p=0.00 respectively). This association remained significant after adjustment for age and BMI.

**Table 1:** Clinical and laboratory characteristics of study participants

		**pGDM N = 77**	**Control N = 67**	**Value**
Age (yr)		32.87 (5.40)	31.66 (5.51)	NS
Income (%)	<600$	77.9	89	NS
	600-900$	18.2	7.5	
	>900$	3.9	3	
Education (%)	Less than diploma	29.9	26.9	NS
	High-school diploma	50.6	53.7	
	College education	19.5	19.4	
Number of delivery (%)	1	40.3	41.8	NS
	2	33.8	34.3	
	3	20.8	22.8	
	4	5.2	1.5	
History of diabetes in family (%)		46.8	13.6	0.00
BMI (kg/m^2^)		28.03 ± 3.61	27.29 ± 5.74	NS
Waist circumference (cm)		91.35 ±9.69	82.84 ± 10.23	0.00
Systolic Blood Pressure (mmHg)		121.35 ± 14.45	108.31 ± 13.16	0.00
Diastolic Blood Pressure (mmHg)		77.19 ± 10.17	73.11 ± 8.90	0.01
FBS (mg/dl)		97.07 ± 15.80	91.19 ± 15.37	0.02
HDL-C (mg/dl)		48.00± 8.57	49.90 ± 8.89	NS
Teiglyceride (mg/dl)		103.28 ± 55.99	123.69 ± 63.01	0.04
Fasting Insulin (mU/ml)		12.01 ± 5.29	7.19 ± 4.62	0.00
hs-CRP (mg/dl) (Median)		2.69 ± 2.86	1.56 ± 1.39	0.00
HOMA-IR		2.88 ± 1.31	1.57 ± 1.02	0.00
LDL-C (mg/dl)		104.09 ± 25.53	87.32 ± 16.81	0.00
IL-6 (pg/ml)		3.56 ± 2.68	2.69 ± 1.77	0.03
Uric-Acid (mg/dl)		4.81 ± 1.12	4.50 ± 1.05	NS
Metabolic Syndrome (%)		9	31.2	0.00
Homocysteine (μmol/dl)		8.19 ± 4.24	8.71 ± 2.35	0.58

Data are expressed as mean ± SD or n (%).

pGDM: Previous Gestational Diabetes, BMI: Body Mass Index, FBS: Fasting Blood Sugar, HDL: High Density Lipoprotein, hsCRP: high sensitive C-reactive Protein, HOMA-IR: Homeostasis Model Assessment–Insulin Resistance, LDL-C Low Density Lipoprotein-Cholesterol, IL-6: Interleukin-6.

Mean CRP levels were different between groups with and without pGDM (2.69 ± 2.86 mg/dl and 1.56±1.39 mg/dl, respectively; p < 0.01 - Table 1). The prevalence of metabolic syndrome in pGDM group was 3 folds higher than the control group (31.2% and 9% respectively; p < 0.01).

In another analysis we compared CRP levels based on individual components of MS in the whole participants of our study free of having MS or not. We found that the presence of each MS component by itself is associated with significantly higher CRP Levels, except for fasting blood glucose (data not shown).

CRP levels were also significantly higher in those with higher BMI (1.33±0.79 mg/dl in those with BMI < 25 kg/m^2^ vs. 2.48±2.82 mg/dl in those with BMI≥25 kg/m^2^; p < 0.01).

**Figure 3:**
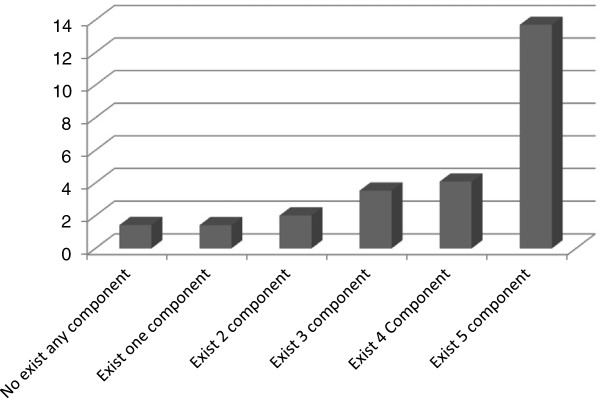
Mean serum level of CRP with increase of MS components.

In next step all participants were divided into 6 groups based on the number of MS components. According to Figure 3, there was a linear increase in CRP levels with an increase in the number of MS components in the group of women with previous GDM. Women with five MS components, have on average, CRP levels four times higher than those with four MS components. In linear regression models, CRP was associated significantly with BMI (β =0.25, p < 0.01), waist circumference (β = 0.27, p < 0.01) and HOMA-IR (β=0.39, p < 0.01). Also the associations between interleukin 6 and BMI, waist circumference and HOMA-IR were assessed (β =0.23, p = 0.05; β =0.39, p < 0.01 and β = 0.58, p < 0.01; respectively).

Then the participants were categorized into 4 groups based on the presence or absence of pGDM and MS criteria. The first group had neither the history of GDM, nor the criteria of MS; the second group had the criteria of MS but not the history of GDM; the third group was vice versa and the last group had both the history of GDM and the criteria of MS. The mean of CRP levels, HOMA-IR indices and serum interleukin 6, FBS, HDL-C and LDL-C levels in these 4 groups are shown in Table 2. There were significant differences in the mean levels of CRP, HOMA-IR, interleukin 6, HDL-C and LDL-C in a univariate ANOVA with Post-Hoc (Scheffe) test. There was a significant difference between the CRP levels of the first group (No GDM/No MS) and the fourth group (who had both history of pGDM and criteria of MS). Although there was also a difference in the mean level of CRP between the first and the third group (No Ms but with GDM), it wasn’t statistically significant (p=0.20). The association between CRP level and pGDM and also MS was assessed using the multiple logistic model in which the first group was set as the reference (Table 3). The analyses revealed that the Odds ratio (OR) of the second group was 1.29; [Confidence interval (CI) =0.43−3.82], the third group was 2.38; [CI=0.39−14.19] and the fourth group was 4.76; [CI=1.54−14.66]. After adjustment for age and BMI, the occurrence of pGDM in the fourth group was significantly associated with CRP level [OR= 5.11; CI = 1.59-16.43].

**Table 2:** Mean of risk factors in each group and comparison of them

	**Group 1**	**Group 2**	**Group 3**	**Group 4**	***p***	Adjusted p*
	**N = 29**	**N = 9**	**N = 83**	**N = 23**		
	**No pGDM no MS**	**MS without pGDM**	**pGDM without MS**	**pGDM with MS**		
CRP mg/dl	1.40 (0.93)	1.97 (1.65)	2.81 (3.03)	4.18 (4.08)	< 0.01	< 0.01
Interleukin 6 mg/dl	2.02 (1.30)	2.30 (1.76)	3.32 (1.82)	6.39 (5.00)	< 0.01	< 0.01
HOMA-IR	1.53 (1.03)	2.41 (0.96)	2.31 (1.12)	4.01 (1.42)	< 0.01	< 0.01
FBS mg/dl	88.29 (11.16)	91.98 (9.77)	95.87 (12.69)	107.64 (20.37)	< 0.01	< 0.01
LDL-C mg/dl	87.40 (17.50)	101.46 (23.05)	86.50 (10.60)	109.56 (29.82)	0.02	< 0.01
HDL-C mg/dl	51.46 (7.95)	51.00 (7.98)	37.75 (6.79)	41.76 (6.13)	< 0.01	< 0.01
BMI kg/m^2^	25.13 (4.35)	27.33 (3.50)	31.39 (4.81)	29.47 (3.49)	< 0.01	< 0.01§
WC cm	81.09 (9.05)	88.44 (8.96)	95.13 (8.23)	97.40 (8.39)	< 0.01	< 0.01

*Adjustment for age and body mass index.

§Adjusted for age.

CRP: C-reactive protein, HOMA-IR: Homeostasis Model Assessment–Insulin Resistance, FBS: Fasting Blood Sugar, LDL-C: Low Density Lipoprotein-Cholesterol, HDL: High Density Lipoprotein-Cholesterol, BMI: Body Mass Index, WC: Waist Circumference.

**Table 3:** Association of high CRP with pGDM and MS in logistic regression model

	**Odds ratio**	**CI of odds ratio**	**p**
**Group 1 (No pGDM no MS)**	1		
**Group 2 (MS without pGDM)**	1.89	0.50-6.19	0.19
**Group 3 (pGDM without MS)**	3.46	0.54-21.93	0.18
**Group 4 (pGDM with MS)**	13.23	3.94 - 44.41	0.00

CRP: C-reactive Protein, pGDM: Pervious Gestational Diabetes, MS: Metabolic Syndrome, CI: Confidence Interval.

## Discussion

Several studies have shown a strong correlation between pGDM and MS. Even Anila Verma *et al*. and Baris Akinci et al. have postulated pGDM as a risk factor for development of metabolic syndrome [21,22]. On the other hand, L. Chatzi and his colleagues have suggested that metabolic syndrome in first trimester could be a risk factor for the development of GDM in late pregnancy [23]. Also in a prospective study by Wolf et al. it was shown that high CRP levels in the first trimester of pregnancy are associated with the increased risk of pGDM, independent from other risk factors such as age, multiparity and smoking [9]. In our research we have tried to explain this discrepancy in means of MS, pGDM and inflammatory markers.

In this investigation the prevalence of MS in women with pGDM was about three folds higher than the control group, which is in concordance with the findings of other studies such as Lauenborg’s [24]. In our study, the mean serum levels of CRP, interleukin 6, HOMA-IR and LDL-C in pGDM women were higher than the control group. Since the increase in these markers is shown to be a risk factor for cardiovascular diseases, this could explain the findings of some studies in which pGDM was defined as a chronic condition that leads to the increased risk of cardiovascular disease [6-8]. Winzer et al. in their study found that in women with pGDM and metabolic syndrome, interleukin-6 level was higher than the group without metabolic syndrome and pGDM [25].

According to the findings of this study, the mean levels of CRP were higher in women with BMI ≥ 25 kg/m^2^ than those who had BMI<25 kg/m^2^. Several epidemiological studies have shown a strong correlation between CRP levels and BMI: the higher the BMI, the higher the level of CRP [26-28]. However, Ferraz et al. could not find this correlation although they found out that the mean CRP levels in women with high waist circumference was significantly higher than those with low waist circumference (with the cut-off of 88 cm) [8].

As we have shown C-reactive protein was not independently correlated with fasting blood glucose levels. This is in accordance with a study in Brazilin women [8]. However, King et al. [29] showed a significant correlation between CRP and FBS. This discrepancy might be ascribed to the differences in methodology, which means the lag time between the survey and delivery, and method of participant selection. For the former reason it is to say that since our examination was carried out earlier than the other studies (12–24 months after delivery compared with 5–11 years after delivery this shorter lag time might have caused this discrepancy. It comes from other studies that there is a correlation between this lag time and the development of diabetes. The longer this period, the higher will be the incidence of type 2 DM [5,30]. Also, diabetic women had been excluded from our study.

As we have shown, the known risk factors of subclinical atherosclerosis or cardiovascular disease such as mean levels of CRP, interleukin 6, HOMA-IR, FBS, and LDL-C were compared between the four groups with following features: the first group: without history of GDM and MS, the second group: women with pGDM but without MS; the third group: women with MS but without the history of pGDM; and the last group: women with both MS and the history of pGDM.

There has also been a positive correlation between the number of MS components and CRP level, so that in those who have all five components of MS, CRP levels are four times higher than those who have only four components of it. This finding is in concordance with that of Ferraz et al. [8]. There were also similar findings about interleukin 6 in our investigation (the results are not shown).

The relationship between inflammatory markers, pGDM and metabolic syndrome has been poorly studied. We could not find any differences in CRP levels between the first group (i.e. without the history of pGDM and MS) and the second group (only with the history of pGDM/without MS). Additionally, there was no significant difference in CRP level of the first and the third group (without MS and history of pGDM, and with MS without history of pGDM, respectively). But the first and fourth group (women with pGDM and MS) were different in terms of CRP level. It means, CRP was higher when the history of pGDM and MS were present compared with those without these two components. The results of this study revealed that in pGDM women who have MS components too, CRP levels increase more than 5 times, compared with women without MS and pGDM. Considering interleukin 6 concentrations between the 4 mentioned groups, it was shown that the mean level of interleukin 6 in the fourth group was about 2 times higher than the third and second groups.

In addition, we found that BMI and particularly waist circumference were associated with CRP levels. This is compatible with studies of other investigators who also found that when the history of pGDM co-exists with MS, there is a synergistic effect on the elevation of serum levels of inflammatory markers. They showed that increased levels of inflammatory markers in MS and pGDM may be related to visceral obesity. Any increase in fat tissue, particularly in abdominal viscera, could lead to the activation of inflammatory cascade [31-34].

## Conclusion

It seems that the presence of either the history of pGDM or MS may result in an increase in inflammatory markers levels such as CRP and interleukin 6, although it was not significant. It should be considered that when these two risk factors are both present they could have a synergistic effect on the rising of inflammatory markers. We could detect association of inflammatory markers with BMI, waist circumference and HOMA-IR as well. Further long-term studies are necessary to confirm the correlation of CRP and MS in women with pGDM and the potential lifestyle and/or pharmaceutical interventions.

## Competing interest

The author(s) declare that they have no competing interests.

## Authors’ contributions

BE drafted the manuscript. HF and AH-N have made substantial contributions to conception and design. HF also revised it critically for important intellectual content. FS has made substantial contribution to the analysis and interpretation of data. ZB and MM have contributed to the acquisition of data. BL has given final approval of the version to be published. All authors read and approved the final manuscript.
